# Prevalence of acquired and transmitted HIV drug resistance in Iran: a systematic review and meta-analysis

**DOI:** 10.1186/s12879-023-08916-3

**Published:** 2024-01-02

**Authors:** Hossein Mirzaei, Sana Eybpoosh, Fatemeh Mehrabi, Mohammad Reza Shojaei, Ali Mirzazadeh, Mehrdad Khezri, Naser Nasiri, Hamid Sharifi

**Affiliations:** 1 HIV/STI Surveillance Research Center, and WHO Collaborating Center for HIV Surveillance, Institute for Futures Studies in Health, Kerman University of Medical Sciences, Kerman, 7616911320 Iran; 2 Department of Epidemiology and Biostatistics, Research Centre for Emerging and Reemerging Infectious Diseases, Pasteur Institute of Iran, Tehran, Iran; 3 Department of Microbiology and Virology, Kerman University of Medical Science, Kerman, Iran; 4 Department of Epidemiology and Biostatistics, Institute for Global Health Sciences, University of California, San Francisco, USA; 5 Department of Epidemiology, New York University School of Global Public Health, New York, NY USA; 6 School of Health, Jiroft University of Medical Sciences, Jiroft, Iran; 7 Affiliate, Institute for Global Health Sciences, University of California San Francisco, San Francisco, CA USA

**Keywords:** Anti-retroviral agents, Drug resistance, Viral, HIV infections, Treatment failure

## Abstract

**Background:**

There is no systematic review on the prevalence of HIV drug resistance (HIVDR) in Iran. We aimed to estimate the prevalence of HIVDR among people living with HIV (PLHIV) in Iran. We assessed HIVDR prevalence in antiretroviral therapy (ART) naïve PLHIV (i.e., those without a history of ART) and PLHIV receiving ART.

**Method:**

We systematically searched Scopus, PubMed, Web of Science, Embase, Iranian databases (Iranian Medical Research Information System, Magiran, and Scientific Information Database), the references of studies, and Google Scholar until March 2023. A random-effects model was used to calculate a point estimate and 95% confidence interval (95% CI) for the prevalence of HIVDR in PLHIV.

**Results:**

Among 461 potential publications, 22 studies were included in the meta-analysis. The pooled prevalence of acquired HIVDR in PLHIV receiving ART was 34% (95% CI: 19, 50) for nucleoside/nucleotide reverse transcriptase inhibitors (NRTIs), 27% (95% CI: 15, 41) for non-nucleoside reverse transcriptase inhibitors (NNRTIs), and 9% (95% CI: 3, 18) for protease inhibitors (PIs). The pooled prevalence of acquired HIVDR in treatment failure PLHIV was 50% (95% CI: 31, 69) for NRTIs, 49% (95% CI: 29, 69) for NNRTIs, 11% (95% CI: 2, 24) for PIs, and 1% (95% CI: 0, 4) for integrase inhibitors (INIs). The pooled prevalence of transmitted HIVDR in ART-naïve people was 3% (95% CI; 1, 6) for NRTIs, 5% (95% CI: 2, 9) for NNRTIs, and 0 for PIs and INIs.

**Conclusion:**

The prevalence of HIVDR was relatively high in both ART-naïve PLHIV and those receiving ART. Without universal pretreatment HIVDR testing and more frequent routine HIV viral load testing among PLHIV who are on ART, the HIVDR prevalence might increase in PLHIV in Iran.

**Supplementary Information:**

The online version contains supplementary material available at 10.1186/s12879-023-08916-3.

## Background

Global access to antiretroviral therapy (ART) has been increased significantly. Based on the World Health Organization (WHO), by the end of 2022, 29.8 million (76%) of the 39 million people living with HIV (PLHIV) were on antiretroviral therapy, and almost 71% of PLHIV had suppressed HIV viral loads [1]. The increased availability and utilization of ART have yielded remarkable outcomes, translating into a remarkable 51% decrease in AIDS-related deaths between 2010 and 2022. This achievement underscores the transformative impact of ART accessibility, marking a substantial stride towards the global goal of ending the HIV epidemic. It exemplifies how strategic efforts to improve access to these life-saving treatments have not only saved lives but have also greatly improved the overall well-being and life prospects of those affected by HIV [1].

However, widespread use of ART has been accompanied by the emergence of HIV drug resistance (HIVDR). HIVDR occurs due to mutation in the genetic structure of HIV viruses that affects the ability of drugs to inhibit the virus replication. These mutations can occur during the viral replication in individuals receiving ART (acquired HIVDR) or when susceptible individuals are infected with drug-resistant viruses (transmitted HIVDR). Transmitted HIVDR is being measured in PLHIV with no history of ART (ART naïve) [2]. HIVDR can threaten the attainment of global targets to end the HIV epidemic. It can reduce the efficacy of drugs, increase the likelihood of death in PLHIV, amplify transmission of HIV to uninfected individuals, and elevate the costs associated with HIV treatment [3]. Monitoring drug resistance patterns and prevalence, either through routine HIV viral load testing or HIVDR testing, is one of the five strategies recommended by WHO to prevent HIVDR [4]. HIVDR testing delivers substantial clinical advantages, aiding in the identification of appropriate drug regimens, ongoing evaluation of treatment effectiveness, prevention of transmission to uninfected individuals, and management of drug resistance. As such, it stands as an indispensable instrument for enhancing the outcomes of HIV treatment [5].

In Iran, an estimated 59,314 people were living with HIV in 2019. Among them, 22,054 individuals (37.2%) were aware of their HIV status, and 14,685 (66.5% of those who were aware of their status) people were on ART [6]. The most common ARTs in Iran are nucleoside/nucleotide reverse transcriptase inhibitors (NRTI), non-nucleoside reverse transcriptase inhibitors (NNRTI), protease inhibitors (PI), and the relatively new integrase inhibitors (INI) [7]. Although HIVDR testing for everyone initiating treatment has demonstrated clinical benefits [8, 9], the HIV treatment program in Iran faces challenges in implementing universal testing for all individuals diagnosed with HIV. The current national guideline recommends HIVDR testing for those who fail to achieve suppressed HIV viral load at six months after starting treatment [7]. However, even within this subgroup, universal HIVDR testing has not been conducted due to cost constraints and limited laboratory capacities [10].

Regularly monitoring drug resistance patterns and prevalence within countries is imperative for effective HIV control and prevention programs. However, established HIVDR testing strategies, and comprehensive national-level surveys on HIVDR are lacking in Iran. Thus, we aimed to conduct a systematic review and meta-analysis to summarize the evidence on HIVDR prevalence among ART-naïve PLHIV and those receiving ART. The findings of this investigation hold the potential to furnish policymakers with data-driven insights for shaping HIV drug policies and initiating periodic national-level surveys.

## Method

This study was conducted following the Systematic Reviews and Meta-Analyses (PRISMA) checklist (Supplementary file S1) and the Peer Review of Electronic Search Strategies. The details of the search, inclusion criteria, and analytic plan are available in the Open Science Framework (osf.io/vxpe5).

### Search strategy

We systematically searched international (including Scopus, PubMed, Web of Science, and Embase) and Iranian databases, including the Iranian Medical Research Information System (https://research.ac.ir/) Magiran (https://www.magiran.com/), and Scientific Information Database: (https://www.sid.ir/) for studies published in English and Persian. We also reviewed the reference list of eligible studies until March 2023. The search terms included (HIV, human immunodeficiency virus, AIDS, acquired immunodeficiency syndrome) AND (antiviral drug resistance, drug resistance, resistance, mutation, drug resistance mutation) were searched in English and Persian. These search terms were combined using appropriate Boolean operators (Supplementary file S2).

### Screening

Following the removal of duplicate citations, studies underwent screening based on their titles and abstracts. The full text of the eligible citations was evaluated for inclusion and exclusion criteria at this stage. The screening process was conducted by two reviewers (HM and FM), with any disagreements resolved through discussion between the two reviewers and consultation with the senior author (HSH).

### Inclusion and exclusion criteria

The included studies met the following criteria: they were community-based cross-sectional or cohort studies assessing the prevalence of HIVDR among PLHIV. Additionally, the studies utilized genotyping methods for evaluating HIVDR and employed the Stanford HIV Drug Resistance Database (http://hivdb.stanford.edu) for resistance assessment. Exclusion criteria comprised studies using data from gene banks or relying on information from medical records of PLHIV, those with unclear patient treatment statuses, studies combining information from treated and untreated patients, case reports or case series studies, and studies reporting data from multiple time points.

### Data extraction

We extracted the following variables from each study: first author, publication year, study period, treatment status (people receiving ART or ART-naïve individuals), study location (city and province), sample size, number of mutations, and type of mutations.

### Risk of bias

The Joanna Briggs Institute’s critical appraisal tool for prevalence studies was used to assess the methodological quality of the included papers. This tool had nine items to evaluate sample size and representativeness of sampling, identification of the condition, description of the study participants, statistical analysis, and managing response rate. If there was a convincing explanation for each of the items in the text of the article, the item was given a score of one and otherwise zero. Therefore, a score between 0 and 9 was given for each article. A lower score meant a higher risk of bias. Two independent reviewers assessed the methodological quality of the included studies (HM and FM). Disagreements were resolved through discussion and consultation with the senior author (HSH) (Supplementary file S3).

### Statistical analysis

Point estimate and 95% Confidence Intervals (95% CI) for the prevalence of HIVDR were estimated for the ART-naïve and people on ART. The Freeman-tukey double arcsine transformation was used to compute the weighted pooled estimate and subsequently reverse-transform it. CIs were computed by employing an equal-tailed test based on the binomial distribution. Heterogeneity between studies was assessed using the I^2^ statistic. Random effect meta-analysis using the DerSimonian-Laird estimate was performed as the I^2^ statistic was more than 50%, representing substantial heterogeneity [11]. Subgroup analysis was conducted based on each drug group (NRTI, NNRTI, PI, and INI). Also, we performed subgroup analysis based on treatment status (all people receiving ART or ART-naïve individuals). The *Metaprop* program was implemented to perform meta-analyses of proportions in Stata 17 [11]. Publication bias was assessed with the funnel plot and Egger's test.

## Results

**Fig. 1:**
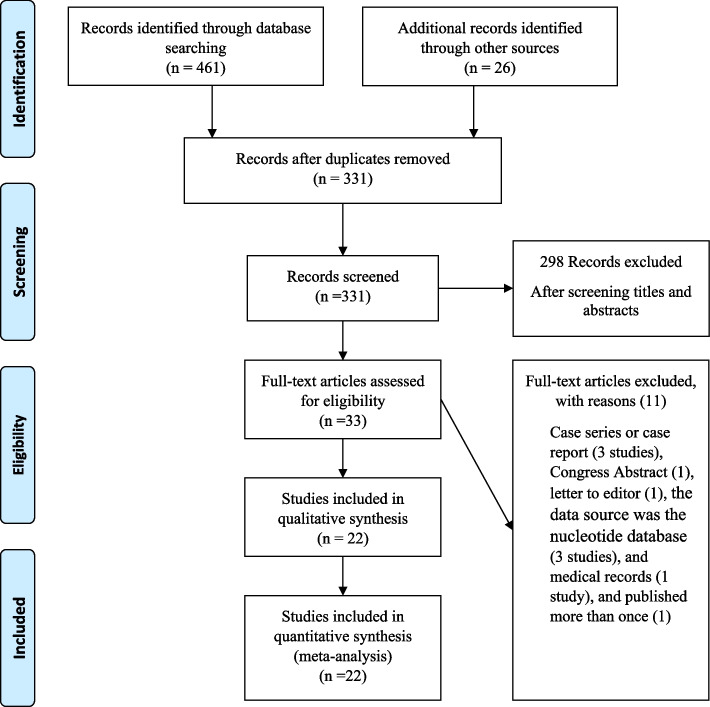
Flowchart of studies included in the systematic review and meta-analysis of HIV drug resistance prevalence in Iran

This review initially identified 487 (461 + 26) potential publications on HIVDR in Iran. After excluding duplicates (156 studies) and unrelated titles and abstracts (272 studies), 33 full texts were evaluated for eligibility. Among these, 11 studies were excluded. Finally, 22 eligible citations were included in the meta-analysis (Fig. 1).

Out of the 22 included studies, nine were conducted on people receiving ART, six on ART-naïve individuals, and seven were on a mixed sample of ART naïve and people receiving ART. Studies were conducted in Tehran (*n* = 9), Bandar Abbas (*n* = 2), Shiraz (*n* = 2), Sanandaj (*n* = 1), Gorgan (*n* = 1), and Ahvaz (*n* = 1). Also, six studies enrolled people from more than one city. All studies were cross-sectional. Fourteen studies focused on the prevalence of HIVDR to three drug groups, including NRTI, NNRTI, and PI, while three studies focused on resistance to PI, one study focused on INI, and one focused on NRTI, NNRTI, and INI. The largest sample size was 655, and the smallest was 25 individuals (Table 1). The quality scores of the included studies ranged from five to nine (out of nine scores) (Table 1, Supplementary file S3).

**Table 1:** Characteristics of studies included in the meta-analysis of HIV drug resistance prevalence in Iran

Study	Study type	Study period	Study group	Province- City	Sample size	Evaluated drugs	Successful sequences﻿	Quality score (out of 9)
Marjani 2020 [12]	Cross-sectional	June 2012 to December 2018	People receiving ART	Tehran	655	Integrase region	62	9
Bokharaei, 2020 [13]	Cross-sectional	April 2013 to September 2018	People receiving ART, ART naïve	Tehran	60 naïve, 592 people receiving ART	NRTI, NNRTI, and PI	60	9
Mohraz, 2019 [14]	Cross-sectional	December 2015 and May 2016	People receiving ART	11 Cities (Tehran, Qom, Esfahan, Gilan, Kermanshah, Ahvaz, Bandar Abbas, Khorramabad, Karaj, Kerman, Hamedan)	207	NRTI, NNRTI, and PI	78	9
Memarnejadian, 2019 [15]	Cross-sectional	April 2016 to March 2017	People receiving ART	Bandar Abbas	44	NRTI, NNRTI, and PI	44	6
Farrokhi, 2019 [16]	Cross-sectional		ART naïve	Tehran, Kermanshah, Esfahan, Shiraz, Mashhad, Gilan, Bandar Abbas, Ahvaz	105	NRTI, NNRTI, and PI	90	7
Nasiri-Tajabadi, 2018 [17]	Cross-sectional		People receiving ART, ART naïve	Tehran, Mashhad	25 25	PI	25 25	6
Memarnejadian, 2018 [18]	Cross sectional	April 2016 and March 2017	ART naïve	Bandar Abbas	41	NRTI, NNRTI, and PI	41	9
Ghafari, 2018 [19]	Cross-sectional	March 2014 to February 2015	ART naïve	Ahvaz	52	NRTI, NNRTI, and PI	52	5
Vahabpour, 2017 [20]	Cross-sectional	September 2015 and July 2016	ART naïve	Tehran	42	NRTI, NNRTI, and PI	42	9
Farrokhi [21]	Cross-sectional		People receiving ART, ART naïve	Tehran	50 28	NRTI, NNRTI, and PI	50 28	6
Naziri, 2016 [22]	Cross-sectional	April 2013 to February 2014	People receiving ART, ART naïve	Shiraz	62 40	NRTI, NNRTI, and PI	62 40	6
Baesi, [23]	Cross-sectional		People receiving ART	Tehran	25	PI	25	5
Memarnejadian, 2015 [24]	Cross sectional	2011	ART- naïve	Sanandaj	40	NRTI, NNRTI, and PI	40	9
Gol Mohammadi, 2015 [25]	Cross-sectional		People receiving ART	Gorgan	130	NRTI and NNRTI	122	6
Baesi, 2014 [26]	Cross-sectional		People receiving ART, ART- naïve	Tehran	70 30	NRTI, NNRTI, and PI	62 62	9
Jahanbakhsh, 2013 [27]	Cross-sectional	January 2010 to February 2011	ART- naïve	Tehran, Kermanshah and Shiraz	50	NRTI, NNRTI, and PI	47	9
Baesi, 2012 [28]	Cross-sectional		ART- naïve People receiving ART	Tehran	30 16	PI	30 15	6
Baesi, 2012 [29]	Cross-sectional		People receiving ART	Tehran	25	NRTI and NNRTI	24	6
Naziri, 2013 [30]	Cross-sectional	Not reported	People receiving ART, ART naïve	Shiraz	20 20	NRTI, NNRTI, and PI	20 20	5
Hamkar, 2010 [31]	Cross-sectional		People receiving ART	Tehran	42	NRT, NNRTI, and PI	42	9
Gholami, 2020 [32]	Cross-sectional		People receiving ART	Tehran, Khorramabad, Qom, Ahvaz, and Hamedan	41	NNRTI, NNRT, PI, and INTI	41	6
Mousavi, 2010 [33]	Cross-sectional		People receiving ART	Tehran, Khorramabad, Ahvaz, Qom, Hamedan	33	NRT, NNRTI, PI, and INIs		9

### Prevalence of acquired HIVDR

**Fig. 2:**
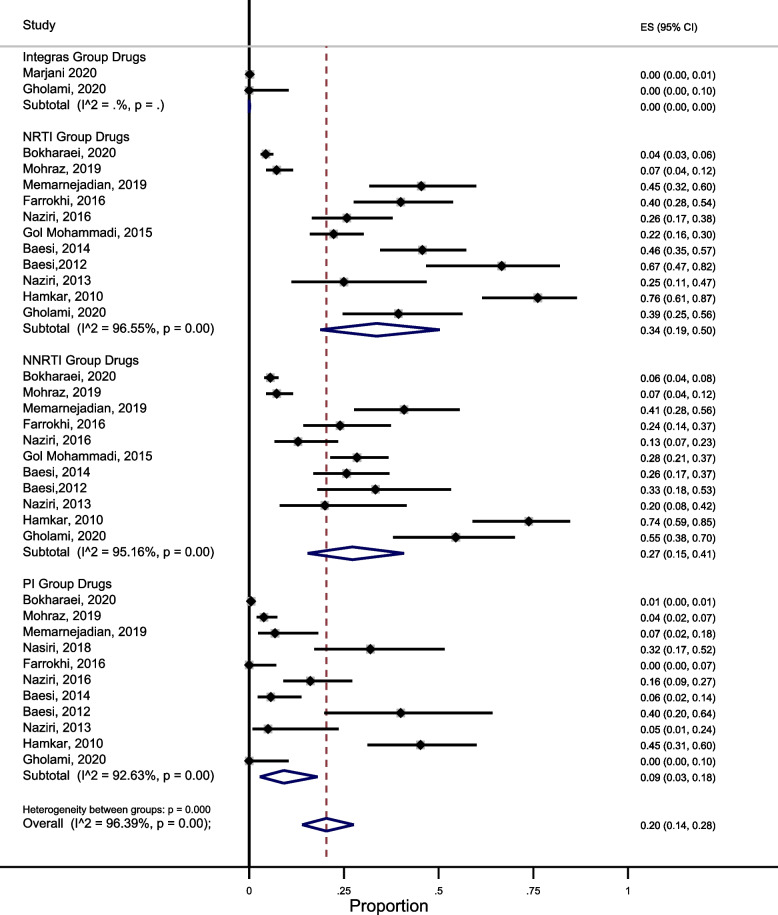
Prevalence of drug resistance in people living with HIV who were under the anti-retroviral treatment in Iran

The pooled prevalence of acquired HIVDR among people receiving ART was 34% (95% CI: 19, 50, I^2^ = 96.55) for NRTIs, 27% (95% CI: 15, 41, I^2^ = 95.16) for NNRTIs, and 9% (95% CI: 3, 18, I^2^ = 92.63) for PIs, and zero for INIs. There was a high degree of heterogeneity among studies in all subgroups (I^2^ = 96.39) (Fig. 2).

**Fig. 3:**
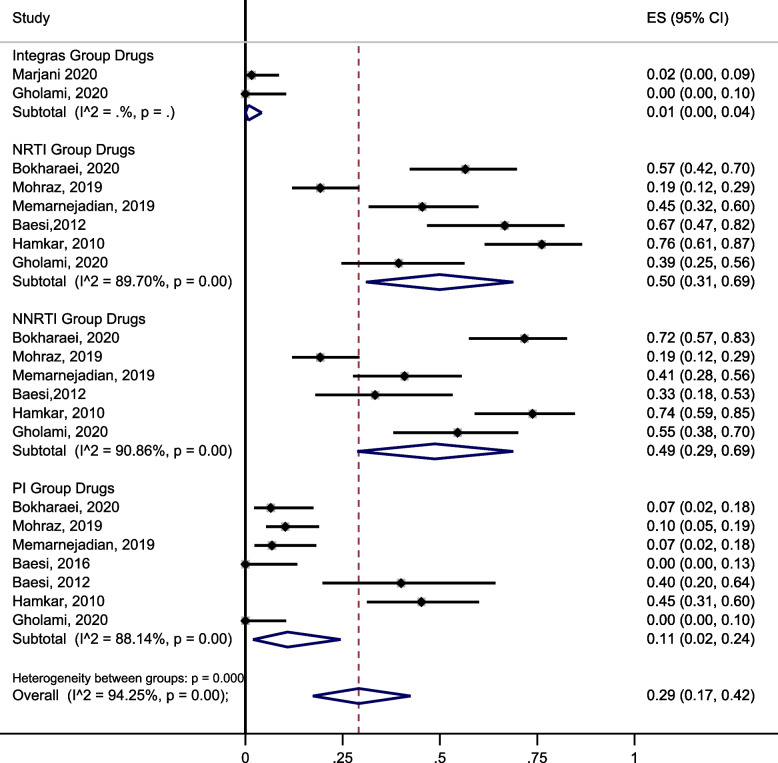
Prevalence of drug resistance in people living with an HIV mutation in one of the drug categories being offered to people living with HIV in Iran

The pooled prevalence of acquired HIVDR in PLHIV with treatment failure was 50% (95% CI: 31, 69, I^2^ = 89.70) for NRTIs, 49% (95% CI: 29, 69, I^2^ = 90.86) for NNRTIs, 11% (95% CI: 2, 24, I^2^ = 88.14) for PIs, and 1% (95% CI: 0, 4, I^2^ = 0) for INIs (Fig. 3).

Among PLHIV with resistance to NRTI drugs, the most common mutations were M184V/I (56.4%), T215Y/N/S/F/I (21.3%), and K219E/Q/R (19.0%). Among the PLHIV with resistance to NNRTI drugs, the most common mutations were K103k/N/S/E (52.2%), P225H (16.1%), and Y181C/S (9.2%). Among the PLHIV with resistance to PI drugs, the most common mutations were V82A/I/M/C (29.0%), M46I (27.4%), and L90M (16.1%) (Table 2).

**Table 2:** Frequency of acquired HIV drug resistance mutations

NRTI Mutations		NNRTI Mutations		Pi Mutations	
Mutation	Frequency (%) of 211	Mutation	Frequency (%) = 184	Mutation	Frequency (%) *N* = 62
M184V/I	119 (56.4%)	K103k/N/S/E	96 (52.2%)	V82A/I/M/C	18 (29.0%)
T215Y/N/S/F/I	45 (21.3%)	P225H	31 (16.1%)	M46I	17 (27.4%)
K219E/Q/R	40 (19.0%)	M230L	17 (9.2%)	L54V/L	11 (17.7%)
V75M/C/A	35 (16.6%)	Y181C/S	17 (9.2%)	L90M	10 (16.1%)
M41L	26 (12.3%)	V108i	15 (8.15%)	L50V/L	9 (14.5%)
D67N/G/Q	23 (10.9%)	K101Ekqr	13 (7.1%)	V32i	6 (9.7%)
K70R/T/I/E/Q	21 (10.0%)	E138A/G	13 (7.1%)	L76v	2 (3.2%)
T69S/N/P/I	11 (5.2%)	V179tf	11 (6.0%)	I84v	2 (3.2%)
K65R/E/I/N/G	11 (5.2%)	K238tn	8 (4.3%)		
F77L/C	5 (2.4%)	A98g	8 (4.3%)		
L74V/I	5 (2.4%)	V106mia	5 (2.7%)		
Y115F	1 (0.5%)	H221Y	3 (1.6%)		
		L100v	2 (1.1%)		
		P236l	1 (0.5%)		
		N348i	1 (0.5%)		

### Prevalence of transmitted HIVDR

**Fig. 4:**
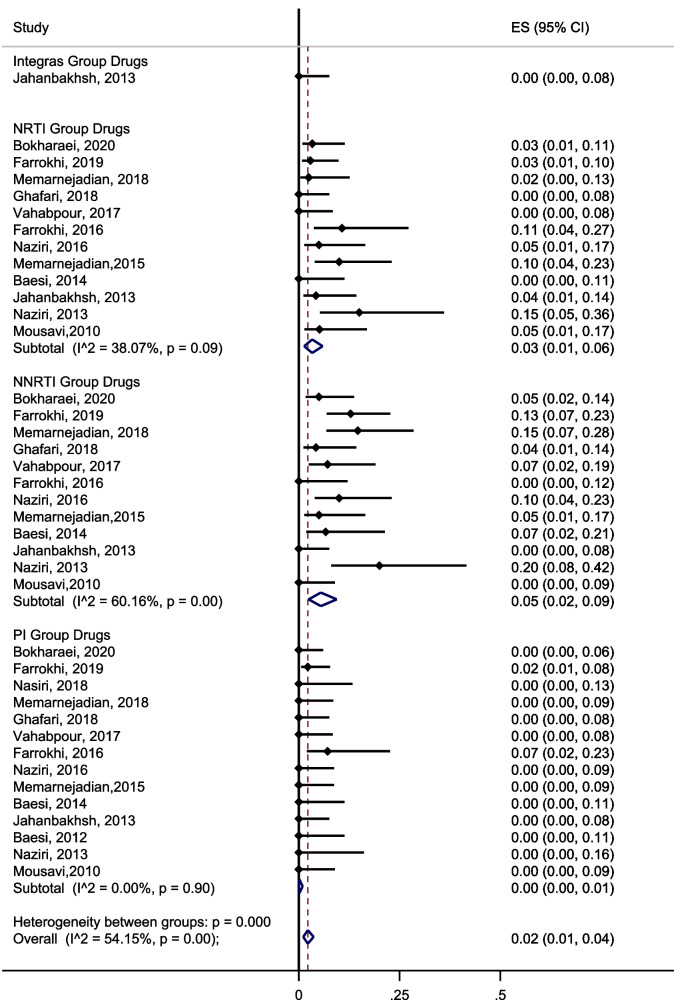
Prevalence of drug resistance among ART-naïve people living with HIV in Iran

The pooled prevalence of transmitted HIVDR in ART-naïve individuals was 3% (95% CI; 1, 6, I^2^ = 38.07) for NRTIs, 5.0% (95% CI: 2, 9, I^2^ = 60.16) for NNRTIs, and 0 for PIs and INIs. There was a medium degree of heterogeneity among studies in all subgroups (I^2^ = 54.15) (Fig. 4).

Among ART-naïve PLHIV with resistance to NRTI drugs, the most common mutations were T215Y/N/S/F/I (55.0%), M184V/I (30.0%), and M41L (25.0%). Among the patients with resistance to NNRTI drugs, the most common mutations were K103k/N/S/E (45.7%), E138A/G (28.6%), and V179T/F (25.7%) (Table 3).

**Table 3:** Frequency of transmitted HIV drug resistance mutation

NRTI related Mutations		NNRTI related Mutations		PI related Mutations	
Mutation	Frequency (%) of 20	Mutation	Frequency *N* = 35	Frequency Mutation	*N* = 4
T215Y/N/S/F/I	11 (55.0%)	K103knse	16 (45.7%)	M46I	2 (50.0%)
M184V/I	6 (30.0%)	E138A/G	10 (28.6%)	L76V	2 (25.0%)
M41L	5 (25.0%)	V179T/F	9 (25.7%)	G48V	2 (25.0%)
D67ngq	4 (20.0%)	V106mia	6 (17.1%)	L50V/L	1 (12.4%)
Y115F	3 (15.0%)	Y181cs	4 (11.4%)		
K65reing	2 (10.0%)	K101Ekqr	2 (5.7%)		
K70rtieq	2 (10.0%)	P225h	2 (5.7%)		
V75M/A/S	2 (10.0%)	K238tn	1 (2.9%)		
L210W	2 (10.0%)				
K219eqr	1 (5.0%)				

**Fig. 5:**
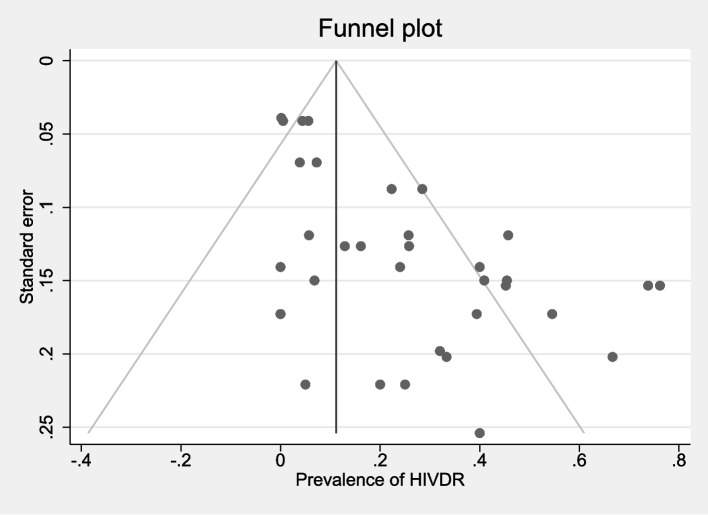
Funnel plot for evaluation of publication bias

The asymmetry in the funnel plots and the result of the Egger test indicated some degree of publication bias (egger test statistic = 5.75, *p* < 0.001) (Fig. 5).

## Discussion

In this systematic review and meta-analysis, we investigated the pooled prevalence of resistance to NRTI, NNRTI, INI, and PI drugs in the ART-naïve and people receiving ART. The pooled prevalence of acquired HIVDR in PLHIV was 34% for NRTIs, 27% for NNRTIs, and 9% for PIs. The prevalence of transmitted HIVDR in naïve PLHIV was 3% for NRTIs, 5% for NNRTIs, and 0 for PIs and INIs.

The pooled prevalence of acquired HIVDR in PLHIV was 34% for NRTIs, 27% for NNRTIs, and 9% for PIs which is higher than the available reports. According to WHO’s 2019 data, the prevalence of any HIVDR among all individuals receiving treatment ranged from 3% in Vietnam to 29% in Honduras [10]. In our study, the prevalence of HIVDR to NNRTIs among populations for whom NNRTI-based first-line treatment failed was 49%. Notably, WHO reported in 2019 that the prevalence of NNRTI resistance in these people ranged from 50% in Eswatini to 97% in Uganda [10]. The pooled prevalence of transmitted HIVDR in ART-naïve individuals was 3% for NRTIs, and 5% for NNRTIs. Based on the WHO report, pretreatment drug resistance to NNRTIs was 7.8% among PLHIV in 18 countries [10].

The higher prevalence of HIVDR in Iran may be attributed to four main reasons. First, there is a low frequency of HIV viral load testing in the country. Studies showed that NNRTI-related HIVDR was higher among individuals who monitored HIV viral load less frequently than every three months compared to more frequently monitored people [34]. National data analysis showed that among 14,685 people on ART in Iran, only 7,471 (50.9%) received HIV viral load testing at least once in 2019 [6]. According to Iran’s national guidelines for HIV treatment, HIV viral load testing is recommended for six months after the initiation of ART for the first time and then every 12 months. Second, the low quality of HIVDR monitoring and surveillance in Iran is a contributing factor. WHO has reported unsatisfactory quality-of-care indicators associated with the emergence of HIVDR in the country. These indicators include retention on ART at 12 months, HIV viral load testing coverage, HIV viral load suppression at 12 months, drug stock-out, and the proportion of people on the second-line ART [10]. Third, there is evidence of low adherence to ART among PLHIV in Iran [35]. Previous studies have reported ART adherence rates ranging from 54.4% to 85.0% [36, 37]. Lastly, the high prevalence of self-medication (i.e., consuming drugs without consulting with a doctor for diagnosis or prescription) in Iran (67%) [38, 39] may contribute to drug-drug interactions, including those with antibiotics, herbal medicine, or even sedatives, further exacerbating the prevalence of HIVDR [40, 41].

The most common NRTI resistance mutation was M184V/I, reducing susceptibility to 3TC (lamivudine)/FTC (Emtricitabine). The most common NNRTI resistance mutation was K103k/N/S/E which reduces susceptibility to NVP (nelfinavir) and EFV(efavirenz); the most common PI resistance mutation was M46I and V82A/I/M/C, which reduces susceptibility to IDV (indinavir), NFV (nelfinavir), FPV, ATV (Atazanavir), and LPV (lopinavir) [42]. The high prevalence of resistance to these drugs can be due to the higher consumption in Iran. The initial drug regimens for PLHIV in Iran before 2020 were two NRTI (Tenofovir + Emtricitabine OR Tenofovir + Emtricitabine OR Tenofovir + Lamivudine) and one NNRTI (Efavirenz). In the latest treatment guideline in Iran, the initial drug regimen changed to one INI (Dolutegravir) and two NRTI. Information about common HIVDR mutations can be used to adopt a new drug as the preferred first-line treatment.

Recognizing the reasons behind HIVDR is pivotal for an effective response to this challenge. In addressing and preventing HIVDR in Iran, a significant step has been taken in the latest national guidelines for the care and treatment of HIV, where the preferred first-line drug for HIV treatment has been switched to Dolutegravir-based antiretroviral regimens [7]. This intervention can help prevent HIVDR because Dolutegravir has a high genetic barrier to resistance [43]. Unfortunately, programmatic quality indicators for HIVDR surveillance in Iran are unsatisfactory [10]. Therefore, emphasizing the standardized HIVDR surveys based on WHO guidelines is necessary. Another strategy for monitoring HIVDR is to add routine pretreatment HIVDR testing in the national HIV program to guide regimen selection [4]. Studies showed that pretreatment HIVDR testing could improve clinical outcomes. This strategy is currently standard practice in high-income countries [44]. While a study in Brazil indicated that pretreatment HIVDR testing is a cost-saving measure [45], findings from a study in Kenya suggested that it may not be cost-effective [46]. The cost-effectiveness of new interventions is contingent on factors such as the prevalence of the related problem, the availability of resources, and established effectiveness thresholds [44]. The absence of evidence regarding the cost-effectiveness of HIVDR testing in Iran underscores the necessity for conducting a comprehensive cost-effectiveness analysis, particularly in the context of pretreatment HIVDR testing.

In addition to treatment, HIVDR can significantly influence the progress of new HIV vaccine development. Vaccines are designed to activate the immune system to identify and combat particular pathogens, such as viruses. However, if the virus has acquired resistance to certain drugs, it may have developed mutations that allow it to evade immune responses triggered by vaccines. These mutations have the potential to diminish the effectiveness of vaccines and curtail their capacity to safeguard against HIV infection [47].

The results revealed a significant level of heterogeneity among the included studies. According to the literature, the primary factors contributing to HIVDR were the specific types of drugs (attributable to variations in genetic barriers) and the treatment status [48, 49]. In subgroup analysis, considering drug categories and treatment status, it was evident that heterogeneity was notably more pronounced among undertreated individuals in comparison to ART-naïve individuals. These findings suggest that one of the principal factors contributing to this heterogeneity may be treatment adherence. It’s important to highlight that we lacked the necessary data to conduct a subgroup analysis regarding this variable. However, previous studies have consistently demonstrated the substantial influence of treatment adherence on the prevalence of HIVDR [48, 50]. Other factors that might contribute to study heterogeneity include transmission routes and specific high-risk groups. Research has demonstrated variations in the prevalence of HIVDr among different risk groups [51].

We found that the distribution of results was asymmetric around the prevalence of 10% in the funnel plot, which indicated that in large studies with small standard error, the prevalence of HIVDR was low, and in small studies the prevalence was high. Furthermore, the results indicated that smaller studies reporting higher prevalence had a greater likelihood of publication compared to smaller studies with lower prevalence. While efforts were made to mitigate publication bias—for instance, searching Iranian databases to address language bias and including conference abstracts and other literature—we acknowledge that complete elimination of publication bias may not have been achieved.

## Limitations

Our study has three main limitations. First, there was a high degree of heterogeneity among included studies. By analyzing data in treatment groups (ART naïve, total people receiving ART, and treatment failure people), heterogeneity decreased in ART-naïve people, while it remained high in people receiving ART. Second, HIVDR in those who were on treatment could have resulted from the transmission of a drug-resistant virus, which we were not able to differentiate. Additionally, when considering HIVDR in individuals initiating ART, it remains unclear whether these ART initiators were genuinely ART-naïve or if participants in the reported studies might have had prior exposure to ART. Third, included studies did not provide information on the prevalence of HIVDR by key populations at high risk for HIV (e.g., female sex workers, people who inject drugs, men who have sex with men).

## Conclusion

Based on the results of this systematic review, the prevalence of HIVDR in ART-naïve individuals and those receiving ART in Iran was relatively high. Without the implementation of universal pretreatment HIVDR testing and more frequent routine viral load testing among PLHIV on ART, these prevalence estimates may continue to rise. The provision of Dolutegravir-based drugs could play a crucial role in preventing the transmission of HIVDR mutations. All of these interventions should also be monitored by a high-quality monitoring and surveillance system.

### Supplementary Information

**Additional file 1: Supplementary file S1.** PRISMA 2020 checklist

**Additional file 2: Supplementary file S2.** Search strategies.

**Additional file 3: Supplementary file S3.** Risk of bias using Joanna Briggs Institute’s critical appraisal tool

## Data Availability

All data generated and analyzed during this study are included in this review.

## Abbreviations

HIVDR: HIV drug resistance
ART: Antiretroviral therapy
PLHIV: People living with HIV
CI: Confidence interval
NRTI: Nucleoside/nucleotide reverse transcriptase inhibitors
NNRTI: Non-nucleoside reverse transcriptase inhibitors
PI: Protease inhibitors
INI: Integrase inhibitors
