# Supplementary material for: Prevalence of acquired and transmitted HIV drug resistance in Iran: a systematic review and meta-analysis
Source: BMC Infect Dis. 2024 Jan 2;24:29. doi: 10.1186/s12879-023-08916-3 (PMC10763184; doi:10.1186/s12879-023-08916-3)
Supplement: Supplementary file 2 — Additional file 2: Supplementary file S2. Search strategies. [file 12879_2023_8916_MOESM2_ESM.docx]

**Supplementary File S2: Search Strategies**

**PubMed: 49 13 march 2023**

((((((((HIV[Title/Abstract]) OR (AIDS [Title/Abstract])) OR (HIV [Mesh])) OR (Human Immunodeficiency Virus [Title/Abstract])) OR (Acquired Immunodeficiency Syndrome [Title/Abstract])) OR (Acquired Immunodeficiency Syndrome [Mesh])) OR (Human Immunodeficiency Virus [Mesh])) AND (((("Drug Resistance, Viral"[Mesh]) OR ("drug resistance"[Mesh])) OR ("mutation"[Mesh])) OR (((((antiviral drug resistance[Title/Abstract]) OR (drug resistance[Title/Abstract])) OR (resistance[Title/Abstract])) OR (mutation[Title/Abstract])) OR (drug resistance mutation[Title/Abstract])))) AND (Iran [Mesh])

**Scopus:145 13 march 2023**

145: ( ( TITLE-ABS-KEY ( human AND immunodeficiency AND virus ) OR TITLE-ABS-KEY ( acquired AND immunodeficiency AND syndrome ) OR TITLE-ABS-KEY ( hiv ) OR TITLE-ABS-KEY ( aids ) ) ) AND ( ( TITLE-ABS-KEY ( drug AND resistance ) OR TITLE-ABS-KEY ( drug AND resistance, AND viral ) OR TITLE-ABS-KEY ( mutation ) OR TITLE-ABS-KEY ( antiviral AND drug AND resistance ) OR TITLE-ABS-KEY ( drug AND resistance AND mutation ) OR TITLE-ABS-KEY ( resistance ) ) ) AND ( TITLE-ABS-KEY ( iran ) )

**Web of science: 120 13 March 2023**

Iran (Topic)

Edit

Query #2

Drug Resistance, Viral (Topic) or drug resistance (Topic) or mutation (Topic) or antiviral drug resistance (Topic) or drug resistance (Topic) or resistance (Topic) or mutation (Topic) or drug resistance mutation (Topic)

Edit

Query #1

HIV (Topic) or AIDS (Topic) or Human Immunodeficiency Virus (Topic) or acquired immunodeficiency syndrome (Topic)

Embase:86

('human immunodeficiency virus infected patient'/exp OR 'aids patient'/exp OR 'acquired immune deficiency syndrome'/exp OR 'acquired immune deficiency syndrome') AND ('iran'/exp OR 'iran' OR 'iran (islamic republic of)' OR 'islamic republic of iran' OR 'persia') AND ('drug resistance'/exp OR 'drug resistance' OR 'treatment failure'/exp OR 'lack of therapeutic efficacy' OR 'therapy failure' OR 'treatment failure' OR 'multidrug resistant infection'/exp)

Iranian databases= 61

other sources=26

461 -156=331
