# Supplementary material for: Prevalence of acquired and transmitted HIV drug resistance in Iran: a systematic review and meta-analysis
Source: BMC Infect Dis. 2024 Jan 2;24:29. doi: 10.1186/s12879-023-08916-3 (PMC10763184; doi:10.1186/s12879-023-08916-3)
Supplement: Supplementary file 3 — Additional file 3: Supplementary file S3. Risk of bias using Joanna Briggs Institute’s critical appraisal tool [file 12879_2023_8916_MOESM3_ESM.docx]

**Supplementary file S3: Risk of bias using Joanna Briggs Institute’s critical appraisal tool**

| **Study ID** | **Sample frame** | **Sampling Method** | **Sample size** | **Description of study subjects and setting** | **Analysis with sufficient coverage of the identified sample** | **Identification of the condition** | **Condition measured in a standard, reliable way for all participants** | **Statistical analysis** | **Response rate** |  |
| --- | --- | --- | --- | --- | --- | --- | --- | --- | --- | --- |
| Marjani,2020 | Yes | Yes | Yes | Yes | Yes | Yes | Yes | Yes | Yes | 9/9 |
| Bokharaei-Salim, 2020 | Yes | Yes | Yes | Yes | Yes | Yes | Yes | Yes | Yes | 9/9 |
| Mohrez,2019 | Yes | Yes | Yes | Yes | Yes | Yes | Yes | Yes | Yes | 9/9 |
| Memarnejadiana, 2019 | No | No | No | Yes | Yes | Yes | Yes | Yes | Yes | 6/9 |
| Farrokhi, 2019 | Yes | No | Yes | Yes | Yes | Yes | No | Yes | Yes | 7/9 |
| Nasiri tajedini, 2018 | No | No | No | Yes | Yes | Yes | Yes | Yes | Yes | 6/9 |
| Memarnejadian, 2018 | Yes | Yes | Yes | Yes | Yes | Yes | Yes | Yes | Yes | 9/9 |
| Ghafari1, 2017 | Yes | Yes | Yes | Yes | Yes | Yes | Yes | Yes | Yes | 9/9 |
| Vahabpour, 2017 | Yes | Yes | Yes | Yes | Yes | Yes | Yes | Yes | Yes | 9/9 |
| Farrokhi, 2016 | No | No | No | Yes | Yes | Yes | Yes | Yes | Yes | 6/9 |
| Naziri, 2016 | No | No | No | Yes | Yes | Yes | Yes | Yes | Yes | 6/9 |
| Baesi | No | No | No | No | Yes | Yes | Yes | Yes | Yes | 5/9 |
| Memarnejadian, 2015 | Yes | Yes | Yes | Yes | Yes | Yes | Yes | Yes | Yes | 9/9 |
| Gol mohamadi, 2015 | No | No | No | Yes | Yes | Yes | Yes | Yes | Yes | 6/9 |
| Baesi, 2014 | Yes | Yes | Yes | Yes | Yes | Yes | Yes | Yes | Yes | 9/9 |
| Jahanbakhsh, 2013 | Yes | Yes | Yes | Yes | Yes | Yes | Yes | Yes | Yes | 9/9 |
| Jahanbakhsh | Yes | Yes | No | Yes | Yes | Yes | Yes | Yes | Yes | 8/9 |
| Baesi, 2012 | Yes | No | No | No | Yes | Yes | Yes | Yes | Yes | 6/9 |
| [Mousavi](https://link.springer.com/article/10.1007/s00705-009-0583-6#auth-Seyed_Mohsen-Mousavi), 2010 | Yes | yes | yes | No | yes | yes | yes | yes | No | 7/9 |
| Gholami, 2020 | Yes | Yes | No | Yes | Yes | Yes | Yes | Yes | Yes | 8/9 |
| Naziri, 2012 | Yes | Yes | No | Yes | Yes | Yes | Yes | Yes | Yes | 8/9 |
| Hamkar, 2010 | Yes | Yes | No | Yes | Yes | Yes | Yes | Yes | Yes | 8/9 |
